# Enhanced Emulsifying Ability of Deoxycholate through Dynamic Interaction with Layered Double Hydroxide

**DOI:** 10.3390/nano13030567

**Published:** 2023-01-30

**Authors:** Jing Xie, Kyounghyoun Lee, Hyeonjin Park, Hyun Jung, Jae-Min Oh

**Affiliations:** 1 Department of Energy and Materials Engineering, Dongguk University, Seoul 04620, Republic of Korea; 2 Department of Chemistry, Dongguk University, Seoul 04620, Republic of Korea

**Keywords:** emulsion formulation, emulsifying ability, emulsifier, deoxycholic acid, layered double hydroxide

## Abstract

The emulsifying ability of the naturally occurring surfactant deoxycholic acid (DCA) was improved by dynamic interaction with nanometric layered particles, layered double hydroxide (LDH). As DCA molecules are rigid due to the facial configuration of hydrophobic–hydrophilic groups, they tend to form molecular aggregation in an acidic condition or imbalanced water–lipid ratios. In this study, the homogeneous hybrids of DCA and LDH were obtained by the in situ growth of LDH at a DCA molecule. The DCA−LDH hybrid successfully prevented the molecular aggregation of DCA at an acidic pH and imbalanced water–to–oil ratio. The dynamic light scattering showed that the hydrodynamic radius of micelle in the emulsion made with DCA−LDH maintained its small size (<500 nm), while upon pH change and dilution with water, that made with DCA only uncontrollably increased up to ~3000 nm. The polydispersity index value of the DCA–LDH emulsion remained constant (<0.3) after the pH change and dilution with water, indicating the high stability of the formulation. Furthermore, time-dependent turbidity monitoring revealed that the DCA-only formulation suffered from serious coalescence and creaming compared with the DCA–LDH formulation. It is suggested that the dynamic interaction between LDH layers and DCA prevented molecular aggregation under unfavorable conditions for the oil–in–water emulsion.

## 1. Introduction

Recent advances in nanotechnology have opened new eras in academic and industrial fields. Scientists intentionally manipulate the atomic arrangement of particles and control the lattice parameters of crystalline materials [1,2,3]. Another group of researchers fine-tune the composition of nanoparticles to control the optoelectronic properties of nanomaterials [4,5]. Nanocomposites prepared in 2-dimensional substrates are often utilized as high-performance biosensors [6,7] or electronic devices [8,9]. Among the various routes to fabricate nanomaterials, the suspension technique is one of the approaches with the most potential in industrial fields, as it can be directly applied to chemical engineering fields. In order to get the homogeneous dispersion of nanoparticles in a solvent media, emulsion technology can be exploited.

Emulsion, that is, colloidal dispersion of two or more immiscible chemical species, has long attracted interest in various industrial fields, such as pharmaceutics, cosmetics, textiles, and agrochemicals [10,11,12,13,14,15]. Specifically, in terms of chemistry, an emulsion is defined as a biphasic system, in which a dispersed phase is homogeneously located throughout a continuous phase. For example, an oil–in–water emulsion, the most widely utilized formulation in cosmetics, has oil droplets as a dispersed phase and water as a continuous phase. However, emulsions are not always thermodynamically stable and tend to be destabilized via physical processes, such as creaming, flocculation, and coalescence. In order to prevent the destabilization of the emulsion, emulsifiers are used to enhance the colloidal stability of the system. The emulsifying ability of emulsifiers refers to the ability to resist a thermodynamically unstable system and enhance kinetic stability [16]. Improvement of the emulsifying ability of emulsifiers is essential to inhibit the destabilization of emulsion [17,18,19]. Surfactant is a representative emulsifier; it both develops a resistant interfacial layer between the dispersed phase and continuous phase, and generates strong repulsive forces among dispersed phases through micelle formation, resulting in the restricted aggregation of droplets [20,21]. Various kinds of emulsifying agents, such as natural (lecithin and Carnauba wax), semi-synthetic (methylcellulose and sodium carboxymethyl cellulose), and synthetic (milk of magnesia, benzalkonium chloride, glyceryl ester, and Tween and Span series) have been developed and utilized in diverse industrial fields. 

In addition to the above-mentioned artificial emulsifiers, naturally occurring amphiphilic molecules, such as deoxycholic acid (DCA), have recently attracted interest due to their low toxicity and wide applicability. DCA is often found in bile acid and is known to be involved in fat dissolution. DCA and bile acids have been researched in various biological fields, such as cholesterol solubilization, dietary fat manufacturing, fat-soluble vitamin absorption, removal of fatty acids from pancreatic hydrolysis, etc. [22,23,24,25,26]. Recently, DCA has been utilized to reduce submental fat [27,28] and to treat obesity-associated fatty liver disease; DCA is one of the most potent inhibitors of the liver-specific fatty acid transport protein 5 [29].

**Figure 1:**
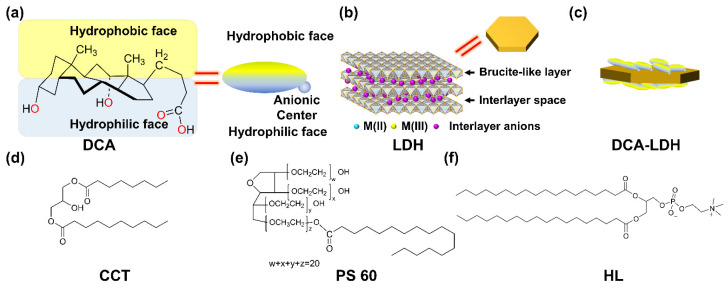
Chemical structures of the materials used in this experimental work: (**a**) sodium deoxycholic acid (DCA), (**b**) layered double hydroxide (LDH), (**c**) DCA adsorbed on LDH (DCA-LDH), (**d**) Caprylic/capric triglyceride (CCT), (**e**) polysorbate 60 (PS 60), (**f**) hydrogenated lecithin (HL).

Despite its performance in biological systems, DCA, as an emulsifying agent, could emulsify immiscible liquids; however, the stability of the emulsion was inferior due to the aggregation of DCA. DCA tends to aggregate, and then it cannot be adsorbed to the interface. Therefore, DCA has limited emulsifying ability under certain chemical conditions. For example, it forms supramolecular aggregates at a low pH, improper temperature, and imbalanced water–to–oil ratio. This can be attributed to the structural limitations of DCA. DCA, as one of the bile salts, is different from a classical surfactant that has a relatively small polar head and a flexible hydrophobic tail. DCA has a facial structure with a hydrophilic (hydroxyl and carboxylate) and a hydrophobic side (steroid ring) (Figure 1a). It has an almost flat shape, with weakly separated hydrophobic and hydrophilic faces. Due to the rigidity in the steroid group, the separation between the hydrophilic and hydrophobic domains is incomplete, and the strong interaction between the hydrophobic parts often results in a formation of aggregates [30,31,32,33]. To the best of our knowledge, no prior report has been made on enhancing the emulsifying ability of DCA under various chemical conditions. One possible way to improve a thermodynamically unstable surfactant-based emulsion was proposed by Nesterenko et al. They conjugated a thermodynamically unstable non-ionic surfactant with hydrophobic silica particles to obtain an increase in critical micellar concentrations and to prevent aggregation at a high concentration [34]. It could be explained that solid particles efficiently stabilized the droplets by forming a network at the interface between two immiscible liquids that acted as a steric barrier against coalescence in the emulsion [35,36]. Nesterenko’s report suggested that the interaction between solid particles and molecular emulsifiers complements the downsides of conventional molecular surfactants.

**Scheme 1:**
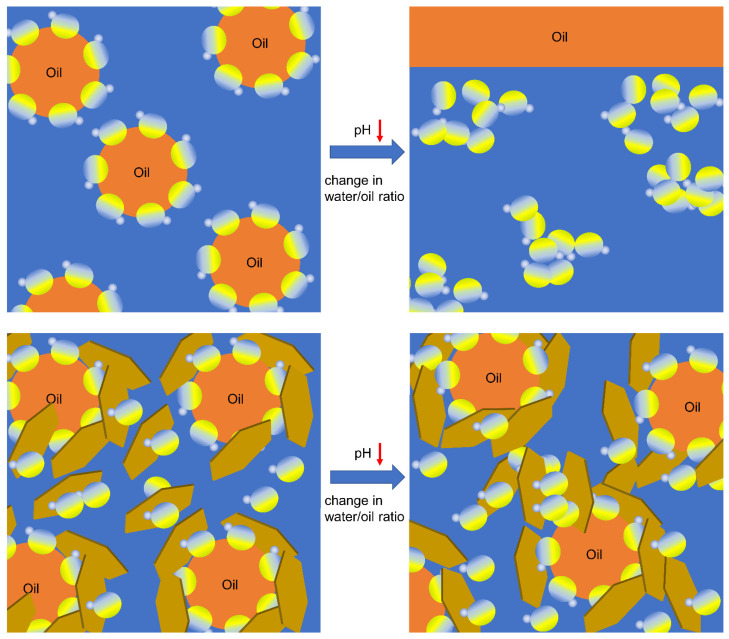
Schematic of the dispersion systems consisting of DCA only or DCA with LDH, before and after stimulation.

Inspired by this approach, we suggest a combination of DCA with nanometric layered particles via electrostatic force. Under aqueous conditions, the DCA molecules adsorb on the nanolayer and detach from the layer very quickly, forming a so-called dynamic equilibrium. Due to the adsorption of the nanometric layered particles, the DCA molecules acquire steric hindrance of the large plate and finally avoid molecular aggregation. We chose layered double hydroxide (LDH), which is a biocompatible anionic clay [37], as the layered nanoparticles (Figure 1b). Since DCA has a carboxylic group as an anionic center, an electrostatic interaction is feasible between DCA and positively charged LDH layers (Figure 1c). In a dispersion, DCA can be adsorbed on the LDH under a dynamic equilibrium. When DCA molecules only are presented in the oil–in–emulsion, they tend to aggregate; however, the existence of a LDH nanolayer, which strongly interacts with DCA, hinders the molecular aggregation and preserves the stable emulsion status (Scheme 1). In order to demonstrate the process in Scheme 1, we designed a relevant hypothesis and experimental sets. The major hypothesis is that the coalescence process can be restrained in the presence of LDH, since LDH particles form a layer surrounding the emulsion droplets. To observe the stability of the emulsion with the assistance of LDH particles, emulsions with either DCA only and DCA with LDH were prepared. Furthermore, the performance of LDH in different conditions was prepared by adding co-emulsifiers, such as polysorbate 60 and hydrogenated lecithin. The emulsions were then treated to unfavorable conditions, such as acid treatment or dilution with water, to show the resistance of LDH toward DCA aggregation. Two main characterizations of dynamic light scattering (DLS) and time-dependent turbidity profiles were carried out to monitor the colloidal behavior of the DCA formulation with and without LDH.

## 2. Materials and Methods

### 2.1. Materials

Sodium deoxycholate (DCA), magnesium nitrate hexahydrate, and aluminum nitrate nonahydrate were purchased from Sigma–Aldrich Co., LLC. (St. Louis, MO, USA). Sodium hydroxide and sodium nitrate were purchased from Daejung Chemicals & Metals Co., Ltd. (Siheung, Gyeonggido, Republic of Korea). Caprylic/capric triglyceride (CCT, PALMESTER3575) was purchased from Palm-Oleo Sdn, Bhd. (Petaling Jaya, Malaysia). Hydrogenated lecithin (HL, Lecinol S10) was obtained from Nikkol Chemicals Co., Ltd. (Tokyo, Japan) and polysorbate 60 (PS 60, Polyoxyethylene(20) sorbitan monostearate, RHEODOL TW-S120V) was obtained from Kao Co. (Tokyo, Japan) All chemicals were of reagent grade and were used without further treatment.

### 2.2. Synthesis of DCA−LDH

Sodium deoxycholate (6.2182 g, 0.015 mol) was added to 100 mL of decarbonated water under continuous stirring for 30 min for dissolution at room temperature (RT), under N_2_ flow; meanwhile, the mixed metal solution was prepared by adding both Mg(NO_3_)_2_·6H_2_O (20.1900 g, 0.07875 mol) and Al(NO_3_)_3_·9H_2_O (9.8450 g, 0.02625 mol) into 100 mL of decarbonated water under continuous stirring for 30 min at RT, under N_2_ flow. Solution (200 mL) was obtained via the mixing of 100 mL of sodium deoxycholate solution and 100 mL of the mixed metal solution at RT, under N_2_ flow. Furthermore, an alkaline solution of 1 mol/L of NaOH was prepared by adding NaOH (9.9992 g, 0.25 mol) to 250 mL of decarbonated water. The suspension was prepared by co-precipitation with increasing pH via adding NaOH at a rate of 50 mL/h. The resulting dispersion was stirred vigorously at RT for 24 h. The precipitates were filtered, washed three times with water, collected by centrifugation at 12,000 rpm (relative centrifugal force, RCF = 6451 g) for 5 min at RT, and then lyophilized for 24 h.

### 2.3. Preparation of the Lipid-Core Capsule Formulations

To prepare the emulsion, caprylic/capric triglyceride (CCT) of a lipid molecule, as a dispersed phase, was selected (Figure 1d). Two kinds of surfactants, polysorbate 60 (PS 60, also known as Tween 60, Figure 1e) and hydrogenated lecithin (HL in Figure 1f), with different hydrophilic–lipophilic balances (14.9 for PS 60, and 9.7 for HL), were chosen as co-emulsifiers to adjust the stability of the emulsions.

All six formulations were prepared by adding raw materials into one reactor and mixing them with a homogenizer. The speed of the homogenizer was slowly increased to 10,000 rpm, and the mixture was thoroughly mixed for 10 min. Table 1 summarizes the weight content of each raw material for the six formulations.

### 2.4. Characterization

Structural characterizations for DCA only and DCA−LDH were carried out with X-ray diffraction (XRD) and Fourier-transform infrared (FT−IR) spectroscopy. The XRD patterns were obtained with Ultima IV (Rigaku, Tokyo, Japan) by utilizing Ni-filtered Cu–Kα radiation (λ = 1.5409 Å, 40 kV, 30 mA) as an X-ray source. The X-ray diffraction patterns were collected in the 2θ range (5−80°), with time-step increments of 0.02° and 0.5 s per step. The FT−IR spectra were recorded using an FT/IR-4600 spectrometer (JASCO, Tokyo, Japan) in the range (2000−600) cm^−1^, with 4 cm^−1^ resolution, to detect transmission at RT. The spectrometer was equipped with an attenuated total reflectance (ATR) accessory and Ge crystal. The powders of DCA and DCA−LDH were placed on the crystal and directly measured via ATR. 

The colloidal stability of the formulations was evaluated by measuring both the hydrodynamic radii and the zeta potentials of the micelles in the diluted conditions, utilizing the zeta potential–particle size analyzer of ELSZ-1000 (Otsuka Electronics Co., Ltd., Osaka, Japan). The intensity-based average diameter (Z-average diameter) and polydispersity index (PDI), which were obtained using a correlation function provided by the TurbiSoft software, were utilized to interpret the hydrodynamic behavior of the formulation. 

The time-dependent turbidity of the formulation against the gravity direction was monitored with Turbiscan Lab Expert equipment (Formulaction Co., Toulouse, France), utilizing static multiple light scattering; the instrument emits a light beam at a wavelength of 880 nm through a cylindrical glass cell containing the sample, and either the transmission or backscattering of light were monitored. For the measurement, emulsion samples were diluted with deionized water to obtain the appropriate turbidity and were placed in a cylindrical glass cell (height = 50 mm). Two synchronous optical sensors could receive the light transmitted through the sample (180° from the incident light), and the light backscattered by the droplets in the sample (45° from the incident light). Both the transmission and backscattering were scanned from the bottom to the top of the emulsion (height over 45 mm), which was repeated every 30 min. The transmission and backscattering were monitored for 7 h at 25 °C.

## 3. Results and Discussion

### 3.1. Structural Analysis of DCA and DCA−LDH

**Figure 2:**
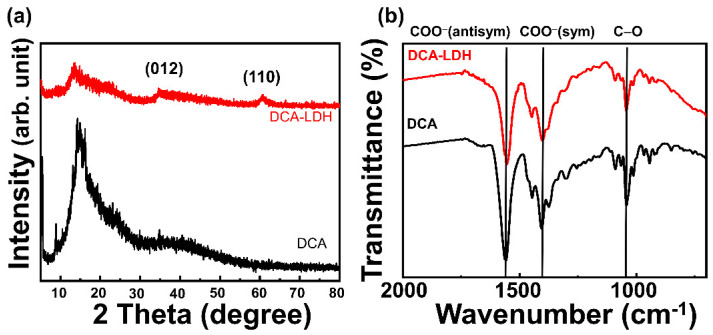
(**a**) X-ray diffraction pattern, and (**b**) FT−IR for DCA and DCA−LDH.

To investigate the degree of interaction between DCA and LDH in the solid state, an X-ray diffraction experiment was first carried out, as shown in Figure 2a. The DCA alone revealed a halo pattern in the range 10° < 2θ < 30° (black line in Figure 2a), indicating the existence of an amorphous arrangement of DCA due to the irregular intermolecular interaction among the sterol moieties through the van der Waals interaction [38]. The XRD pattern of DCA−LDH also showed a broad, amorphous pattern; however, the degree was much less than in DCA alone (Figure 2a). It was noteworthy that we could observe the development of peaks at 34.4° and 60.7°, which can be indexed to the (012) and (110) crystal planes, respectively, of LDH, according to the Joint Committee on Powder Diffraction Standards (JCPDS) 22-0700 (red curve in Figure 2a) [39,40]. 

Different from the conventional LDH, (00l) patterns, the characteristics of 2-dimensional materials, were not observed; this absence was attributed to the poor ordering of the layer stacking along the c-axis. The existence of asymmetric lattice peaks for (012) strongly indicated the formation of a turbostratic-structured LDH phase, corroborating the poor ordering of the c-axis stacking. We could obtain two major points in the XRD pattern of the DCA−LDH: (i) the intermolecular interaction in DCA was strongly inhibited, and (ii) the crystallite size of the LDH particle was sufficiently small, and the layer stacking was sufficiently disordered to facilitate interaction with the molecular DCA. In other words, both the DCA and LDH moieties were homogeneously blended at the nanometer scale to block assembly between the same species. Supposing that this interaction existed in the dispersion, the molecular aggregation of DCA could be substantially prevented. 

Further information on the structure of DCA−LDH was examined with FT−IR spectra, as shown in Figure 2b. Both spectra of DCA and DCA−LDH showed similar patterns, displaying bands for the stretching vibration of the C−O bond (1042 cm^−1^) [41,42], and antisymmetric and symmetric COO^−^ stretching (at around 1550 and 1400 cm^−1^, respectively). The results confirmed that the deoxycholate was well preserved after adsorption onto the layered particle surface, which is attributed to the electrostatic interaction between the carboxylate moiety of the DCA and the positive charge of the LDH layer [43]. Both XRD and FT−IR analyses revealed that the DCA molecules could be bound to the LDH layer in the solid state, and that the DCA would have affinitive interaction with LDH, even in the suspension state. Although the adsorbed DCA can be detached from LDH under aqueous formulation, we are confident that the overall DCA−LDH is maintained through dynamic equilibrium.

### 3.2. Stability of DCA- and DCA−LDH-Containing Formulations in Acidic Condition

**Figure 3:**
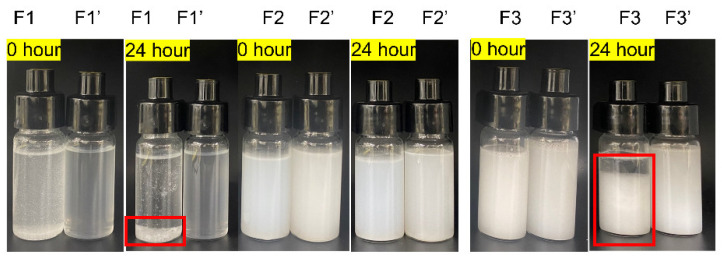
Photos of the different formulations at lowered pH (~5.5) for 24 h.

The stability of formulations at lowered pH (~5.5), depending on the type of emulsifier (DCA or DCA−LDH), was monitored by photographs for 24 h (Figure 3). The critical micellar concentration (CMC) of DCA is known to be ~0.1 wt% in water at 25 °C [44], and it could form aggregated liquid crystal at an elevated concentration. As the concentration of DCA in this study is 1%, we are fairly sure that the DCA would form micelle in the formulation and that it would aggregate under an imbalanced condition. According to the previous report, inorganic particles, which can adsorb surfactants, tended to reduce the CMC of surfactants [45]. Therefore, we expected that the CMC of DCA could be modified to a lower value and the critical concentration to form aggregates would be dropped down. 

It was clearly shown that F1 and F3 (DCA formulations) resulted in coalescence and sedimentation, respectively, as identified by the red rectangles in the figure, suggesting that at slightly acidic pH, oil droplets gathered rapidly due to the aggregation of DCA moiety. The stabilities of the corresponding DCA−LDH formulations (F1′ and F3′) were apparently higher than those of the DCA ones. This indicated that when DCA interacted with LDH, the intermolecular aggregation of DCA in micelles could be effectively inhibited. This may be due to the steric hindrance of nanometer-sized particles to block the molecular aggregation of DCA. In order to solve the aggregation of DCA, Zhang et al. recently combined DCA with silica particles and achieved high stability of an octane–water emulsion, even at a high concentration [46]. A similar approach to enhance emulsion stability with inorganic particles was reported by Bhatia’s group [47]. They combined clay particles with surfactants of sodium dodecyl sulfate, Pluronic F68, and dodecyltrimethylammonium bromide to prepare an oil–in–water emulsion. They found that the zeta potential change initiated by the clay particles altered the characteristics of the emulsions. Although it is not clear whether the action of the silica or clay was similar to that of the current LDH, we are fairly sure that the existence of inorganic particles influenced the aggregation of DCA molecules in an unfavorable condition.

We could not observe clear differences of stability between F2 and F2′, possibly due to the strong stabilizing effect of PS 60, which has a high hydrophilic–lipophilic balance (HLB = 14.9). The detailed difference in the colloidal stability of the DCA formulation, with and without LDH moiety, is quantitatively investigated below by dynamic light scattering and time-dependent turbidity change.

### 3.3. Colloidal Behavior of DCA- and DCA−LDH-Containing Formulations

**Figure 4:**
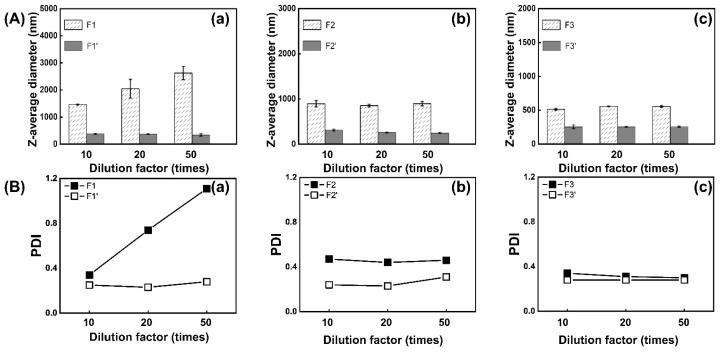
(**A**) Z-average, and (**B**) polydispersity index (PDI, plotted as black squares), of emulsions with respect to dilution factor for (**a**) F1 and F1′, (**b**) F2 and F2′, and (**c**) F3 and F3′.

To investigate the colloidal stability of DCA in emulsions, its average hydrodynamic radius (Z-average) and polydispersity (PDI) were monitored under an imbalanced water–to–oil ratio by diluting it with 10-, 20-, and 50-times deionized water (Figure 4, and Table S1 of the Supplementary Materials). Notably, the Z-averages of emulsions in the presence of DCA−LDH (F1′, F2′, and F3′) did not change significantly upon dilution up to 50 times, preserving the emulsion size at less than 500 nm (Figure 4A). The droplet size is important for the stability of the emulsion. On the other hand, the emulsion with DCA only (F1, F2, and F3) readily destabilized, showing a significantly higher Z-average than DCA−LDH emulsions (F1′, F2′ and F3′). Apparently, when the water–to–oil ratio changed, the aggregation among DCA molecules was facilitated, while when LDH nanolayers co-existed with DCA, molecular aggregation was strongly prohibited. This could be interpreted as the DCA−LDH-containing emulsions being well dispersed in the highly diluted condition. The difference in colloidal stability between the DCA emulsion and the DCA−LDH emulsion was most clearly shown in the F1 and F1′ pair. The other two pairs (F2/F2′ and F3/F3′) did not suffer severe aggregation, possibly due to the presence of a co-emulsifier, such as PS 60 or HL. 

In addition to the Z-average values of emulsions, the PDI also gave meaningful information on the stability of an emulsion. The PDI value is the ratio between the square of the standard deviation and the average value; when the value is below 0.3, it indicates monodispersity [48,49]. As shown in Figure 4B, the PDI values of the DCA−LDH emulsions (F1′, F2′, and F3′) were below 0.3, while those of the DCA emulsions (F1, F2, and F3) were higher than 0.3. It is worth noting here that F1, which contained DCA as the only emulsifier, exhibited a serious increase in PDI value; the value was 0.34 at 10-times dilution, but it increased to around 1.00 at 50-times dilution. It is well known that DCA, as a component of bile salt, can emulsify large fat droplets into small ones [50,51]. However, we observed that the increased amount of water could disturb the action of DCA as an emulsifier, resulting in the growth of fat droplet size. From the dynamic light scattering study, we could suggest that when LDH particles existed near the DCA molecules to interact with them dynamically, the emulsifying ability of DCA could be preserved.

### 3.4. Zeta Potential of DCA in Oil–in–Water Emulsions

**Figure 5:**
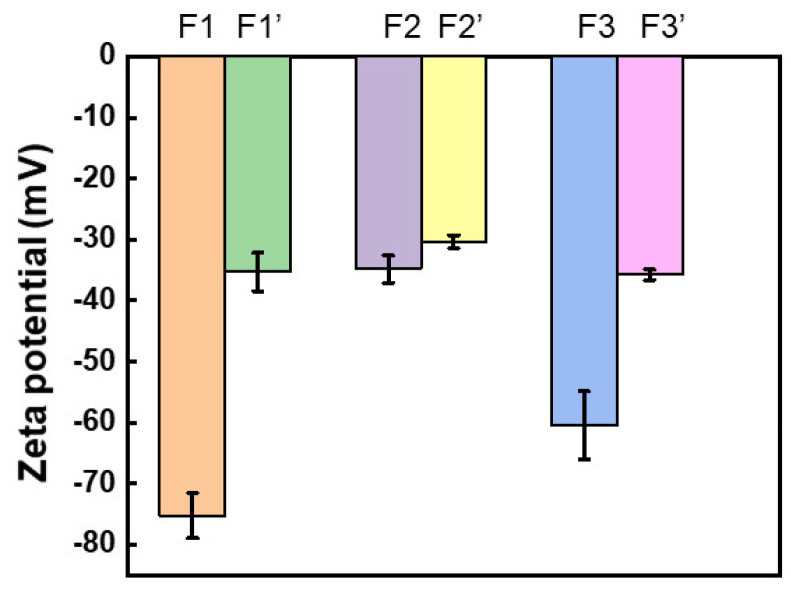
Histograms for zeta potentials of the prepared emulsions: F1, F1′, F2, F2′, F3, and F3′.

The zeta potential, one of the parameters to estimate the stability of emulsion by electrostatic repulsion, was monitored to determine the colloidal stability of the emulsions (Figure 5, and Table S2 of the Supplementary Materials). The absolute values of zeta potential for all emulsions were greater than 30 mV, indicating that all the emulsions had sufficient repulsive force among particles to attain colloidal stability [52]. Even the absolute value was higher for DCA emulsions (F1, F2, and F3) than for DCA−LDH emulsions (F1′, F2′ and F3′). This conflicts with the hydrodynamic radius data in Figure 4A, which showed more stabilized colloids for less charged emulsions. The less negative charge of DCA−LDH can be interpreted as the charge neutralization of negative DCA by positive LDH [53,54]. Due to the positive layer charge and high surface area, LDH has a high affinity to anionic species through electrostatic interactions [55,56]; thus, various negatively charged species can be adsorbed on the surface of LDH layers through electrostatic interactions. As deoxycholate is an anion, it also adsorbs on the surface of LDH layers [57]. When the DCA molecules (negative charge) are adsorbed on the LDH surface (positive charge), the negative charge of DCA would be camouflaged by the LDH moiety; vice versa, the positive charge of LDH would also be screened by the DCA moiety. In this way, both DCA and LDH would lose their intrinsic negative and positive charge to make neutralization. Even with less negative charge, the LDH particles interacting with DCA hindered the intermolecular aggregation of DCA to reduce the overall hydrodynamic size. The DCA micelle did not avoid fusion among the micelles under imbalanced water–to–oil ratio; this may be due to the strong intermolecular aggregation under high water content, which exceeds electrostatic repulsion. On the other hand, the DCA moieties in DCA−LDH micelles are under dynamic equilibrium with LDH layers; thus, the molecular aggregation was prevented, regardless of the water–to–oil ratio. These results suggest that LDH provided a good solid platform to stabilize the emulsifying ability of DCA. Another point to be noted in the zeta potential values of DCA micelles is the significant disparity with respect to co-emulsifier. As clearly shown in Figure 5, the values of F1, F2, and F3 were −75, −35, and −65 mV, respectively. This indicates that the DCA emulsion was highly dependent on the action of co-emulsifiers [58,59] due to their intrinsic instability arising from the intermolecular aggregation. However, the F1′, F2′, and F3′ emulsions did not show a difference in zeta potential, regardless of co-emulsifier, which was attributed to the major stabilizing effect of the LDH over other co-emulsifiers.

### 3.5. Stability Monitoring by Time-Dependent Turbidity

**Figure 6:**
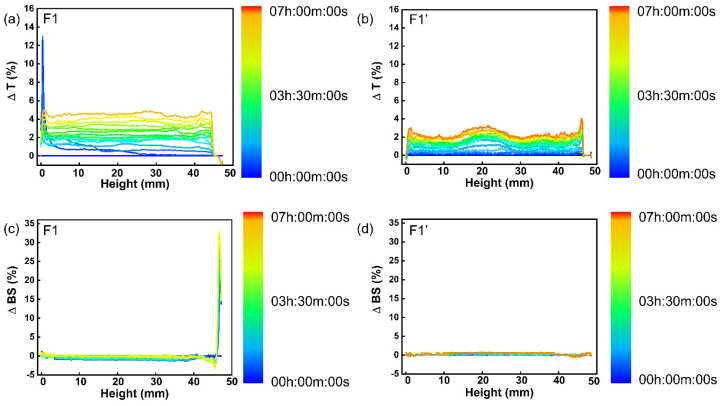
Turbiscan profiles of (**a**,**b**) transmission, and (**c**,**d**) backscattering, for F1 and F1′ over 7 h.

In addition to the dynamic light scattering and zeta potential measurement, the stability of emulsions was investigated by multiple light scattering using TurbiscanLab^®^ equipment. Figure 6 shows the time-dependent change in transmission (ΔT) and backscattering (ΔBS) at the various height positions of the vial (50 mm in height) that was monitored. We could observe a slight increase in ΔT for both F1 (Figure 6a) and F1′ (Figure 6b), which indicated that both emulsions underwent coalescence to some extent, giving rise to inter-particle space for light transmission. However, note here that the degree of ΔT was higher in F1 than in F1′ over 7 h, suggesting less tendency of coalescence in F1′ than in F1. In addition, F1 showed a sharp increase (>12%) in ΔT at an early time in the bottom part (0−5 mm in height), which clearly indicated a decrease in particle concentration by coalescence or Ostwald ripening [60,61,62]. As we could not observe these phenomena in F1′, we could expect the higher stability of the DCA−LDH emulsion than of the DCA one. 

The backscattering signal of both emulsions showed almost zero in the vial height range 0−45 mm (Figure 6c,d). Although zero ΔBS indicates negligible particle growth or aggregation, we could not ascertain the stability of either emulsion due to the low concentration of the tested samples. Therefore, we focused on the dramatic increase of ΔBS in height >45 mm for F1 (Figure 6c). The increased ΔBS at the top side of tube could be accounted for by the creaming phenomenon, which occurred in the gathering of creamed emulsion at the top. Creaming is the instinctive tendency to form a concentrated cream layer at the top of an oil–in–water emulsion. If there is a segregation of oil and water, the oil droplets will cream up due to the density difference [63]. The ΔBS results suggested that oil droplets in DCA-only emulsions migrated to the top as the DCA lost its emulsifying ability, resulting in the segregation of the oil and water phases. Although DCA only has been reported to stabilize the oil phase by itself [46,64,65,66], the current finding indicates that the emulsifying capacity of DCA was limited to a certain pH range and balanced water–to–oil ratio. However, the emulsifying ability could be improved by interaction with LDH. The dynamic interaction between DCA and LDH greatly suppressed the molecular aggregation of DCA; thus, the formulation with DCA−LDH acquired stability against aggregation.

It can be generally expected that the low surface energy of the micelle increases the stability of micelles by avoiding interarticular fusion. According to the previous report, the interfacial tension increased after the addition of plate-like particles into the water–in–oil emulsion. The adsorption of surfactants on the surface of the particle led to a decrease in surfactant density, increasing interfacial tensions. In spite of the increased surface tension, the paper revealed that the colloidal stability increased under the existence of plate-like particles, possibly due to the network formed by the large particles [67]. Similarly, we believe that the colloidal stability was not only governed by the interfacial tension, but was also influenced by the physical interaction between additives and the micelle surface. Similarly, the DCA–LDH formulation, which would apparently have higher surface tension than the DCA-only formulation, could acquire higher colloidal stability.

**Figure 7:**
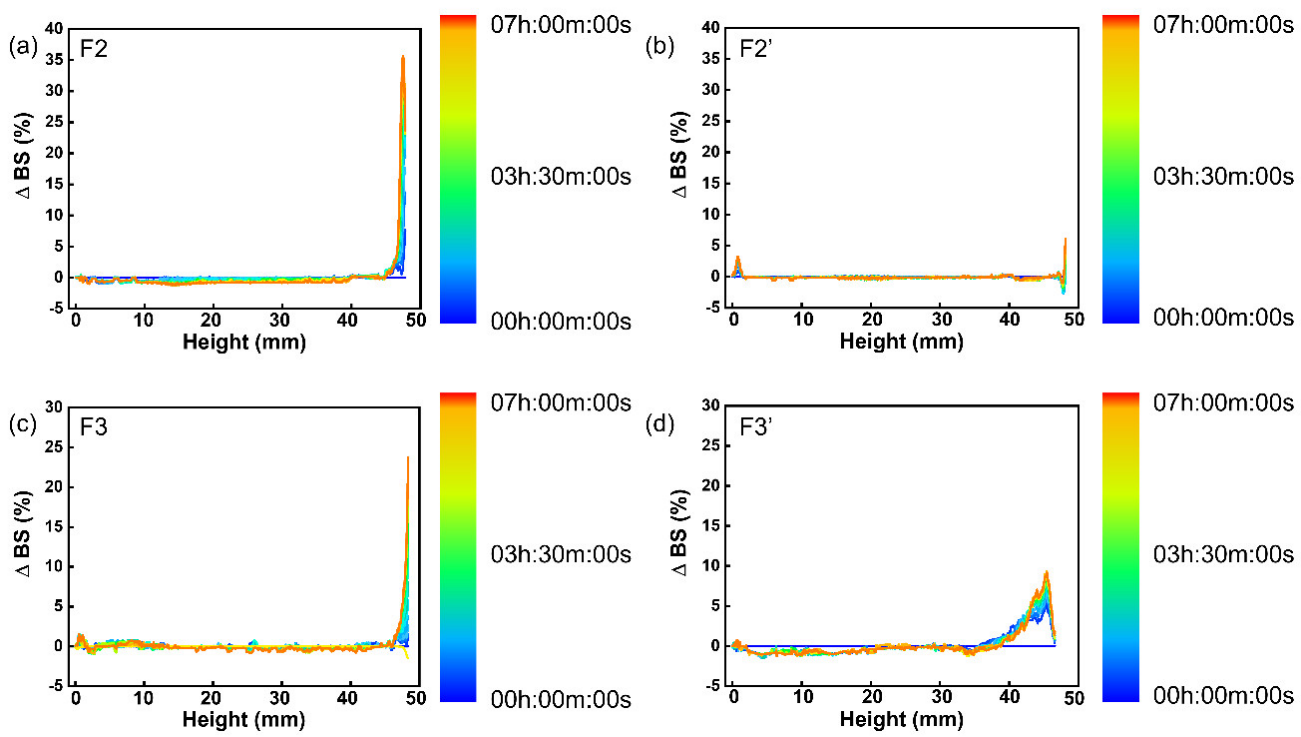
Turbiscan profiles of backscattering for (**a**) F2, (**b**) F2′, (**c**) F3, and (**d**) F3′, over 7 h.

The stability of emulsions in the presence of co-emulsifiers, i.e., PS 60 or HL, was also monitored and is displayed in Figure S1 of the Supplementary Materials (ΔT) and Figure 7 (ΔBS). After 7 h, we could not observe any significant change in ΔT due to the excellent stabilization ability of PS 60 and HL (Figure S1 of the Supplementary Materials). The difference between DCA and DCA−LDH was found at the top of the tube (height > 40 mm) in ΔBS. For example, ΔBS at the top of F2 was ca. 35%, indicating particle migration to the top of the sample tube, i.e., creaming [61,68]. On the other hand, a slight ΔBS increase (~6%) in F2′ indicated less tendency of particle movement, suggesting the good stabilization system of the F2′ formulation. This is consistent with the DLS data, where the mean particle sizes of the F2′ formulation were smaller than that of F2 (shown in Figure 4). The situation was similar to the formulations with the HL co-emulsifier; it was clearly observed that an increase of ΔBS at the top of the tube was much higher for F3 (25%) than for F3′ (10%), representing that there was a larger tendency of creaming in the F3 than the F3′ formulation [69,70]. The result corroborated the excellent property of DCA−LDH as an emulsifier.

## 4. Conclusions

In conclusion, the stability of DCA emulsions was improved with the assistance of LDH, even at a low pH and imbalanced water/oil ratio. Dynamic light scattering clearly showed that the hydrodynamic radius of the DCA-only emulsions increased dramatically at a low pH and high water–to–oil ratio, while the formulations made with DCA−LDH were not affected in terms of hydrodynamic size, even in an acidic condition and 50-times dilution with water. The PDI value of the DCA–LDH emulsions remained fairly constant under 0.3 after pH change and dilution with water. The sizes of oil droplets in DCA–LDH emulsions (<500 nm) were relatively small compared to DCA-only emulsions (much larger than 500 nm) with the same oil content. These results suggested that the presence of LDH particles resists emulsion coalescence, possibly through surrounding the emulsion droplets with LDH layers. The zeta potential of DCA−LDH micelles was less negative than that of DCA ones, indicating that LDH particles closely attach to the droplet surfaces by dynamic interaction toward DCA. The multiple light scattering, i.e., time-dependent turbidity, presented that the change in transmission of emulsions stabilized by DCA−LDH (ΔT < 4%) was smaller than that of emulsions made with DCA (ΔT > 12%), suggesting the superior stability of the DCA−LDH-containing emulsion over the DCA-containing ones. With the assistance of LDH, DCA acquired high dispersibility in emulsions and avoided molecular aggregation. The emulsions with small droplet sizes have high applicability in various biological fields, including drug delivery systems and nutraceutics.

## Figures and Tables

**Table 1:** Content (in % wt/wt) of DCA or DCA−LDH; CCT; and co-emulsifier in each formulation.

Formulation	DCA	CCT	Co-Emulsifier	Water	Formulation	DCA−LDH	CCT	Co-Emulsifier	Water
F1	1%	2%	-	97%	F1′	2%	2%	-	96%
F2	1%	2%	PS 60 2%	95%	F2′	2%	2%	PS 60 2%	94%
F3	1%	2%	HL 2%	95%	F3′	2%	2%	HL 2%	94%

## Data Availability

The data presented in this study are available upon request from the corresponding author.

## Supplementary Materials

The following supporting information can be downloaded at: https://www.mdpi.com/article/10.3390/nano13030567/s1, Figure S1: Turbiscan profiles of transmission for (a) F2, (b) F2′, (c) F3, and (d) F3′, over 7 h; Table S1: Z-average diameters and Polydispersity indices (PDI) of emulsions with respect to dilution factor of (10, 20, and 50) times for F1, F1′, F2, F2′, F3, and F3′; Table S2: Zeta potentials of emulsions diluted by 10 times for F1, F1′, F2, F2′, F3, and F3′.
