# Supplementary material for: Enhanced Emulsifying Ability of Deoxycholate through Dynamic Interaction with Layered Double Hydroxide
Source: Nanomaterials (Basel). 2023 Jan 30;13(3):567. doi: 10.3390/nano13030567 (PMC9920619; doi:10.3390/nano13030567)
Supplement: Supplementary file 1 [file nanomaterials-13-00567-s001.zip › nanomaterials-2157499-supplementary.pdf]

Supplementary Information

Enhanced Emulsifying Ability of Deoxycholate through Dynamic Interaction with Layered Double Hydroxide

Jing Xie [1,†], Kyounghyoun Lee [2,†], Hyeonjin Park [1], Hyun Jung [2,*] and Jae-Min Oh [1,*]

[1] Department of Energy and Materials Engineering, Dongguk University, Seoul, 04620, Republic of Korea
[2] Department of Chemistry, Dongguk University, Seoul, 04620, Republic of Korea
* Correspondence: chemphile@dongguk.edu (H.J.); jaemin.oh@dongguk.edu (J.-M.O.)
† These authors contributed equally to this work.

1. DLS

Table S1. Z-average diameters and Polydispersity indices (PDI) of emulsions with respect to dilution factor of (10, 20, and 50) times for F1, F1', F2, F2', F3, and F3'.

Sample	Z-Average Diameter (nm)			Polydispersity Indices		
	**10**	**20**	**50**	**10**	**20**	**50**
F1	1,450.0	2,036.3	2,615.0	0.34	0.74	~ 1.00
F1'	363.9	355.7	322.0	0.25	0.23	0.28
F2	885.8	844.6	885.9	0.47	0.44	0.46
F2'	302.7	248.2	235.1	0.24	0.23	0.31
F3	508.4	551.4	549.9	0.34	0.31	0.30
F3'	248.2	248.5	249.5	0.28	0.28	0.28

2. Zeta potentials

Table S2. Zeta potentials of emulsions diluted by 10 times for F1, F1’, F2, F2’, F3, and F3’.

Sample	Zeta Potential (mV)
F1	−75.27 ± 3.71
F1’	−35.31 ± 3.16
F2	−34.84 ± 2.22
F2’	−30.33 ± 1.07
F3	−60.42 ± 5.59
F3’	−35.75 ± 0.83

3. Turbiscan profiles

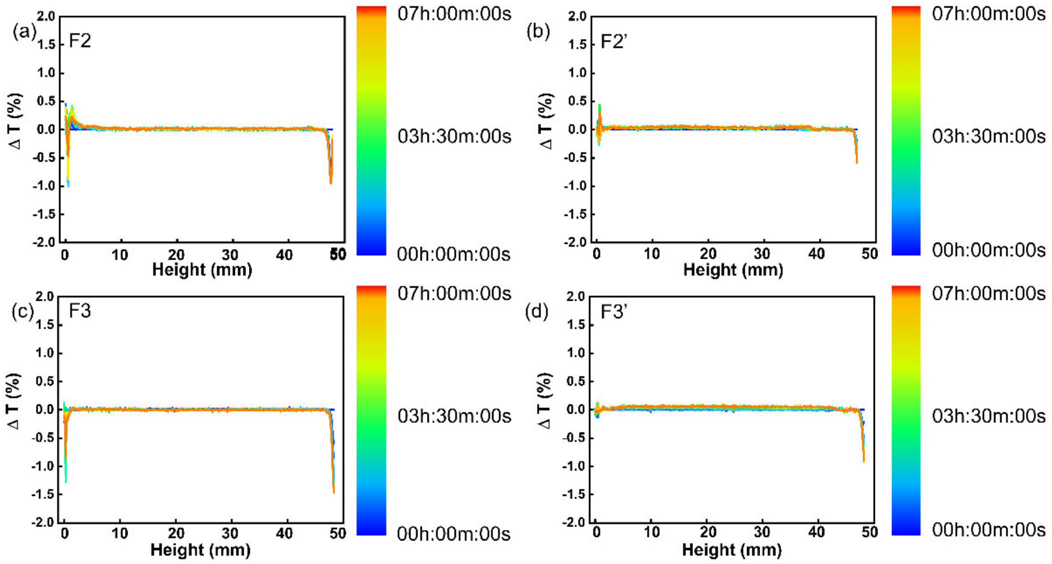

Figure S1. Turbiscan profiles of transmission for (**a**) F2, (**b**) F2’, (**c**) F3, and (**d**) F3’, over 7 hours.
